# Incidence and factors associated with bronchopulmonary dysplasia: a cohort study in a high-risk Colombian neonatal intensive care unit

**DOI:** 10.1590/1984-0462/2026/44/2026003

**Published:** 2026-09-21

**Authors:** Andrea Jaramillo-Cerezo, Sofía Castrillón-Acosta, Daniela Ortiz-Piedrahita, Laura Rodriguez-León, María Camila Paniagua-López, Libia Rodriguez-Padilla, Natalia Pérez-Doncel, Wendy Meyer Martínez, Andrea Parra-Buitrago

**Affiliations:** a Universidad Pontificia Bolivariana, Medellín, Antioquia, Colombia.; b Clínica Universitaria Bolivariana, Medellín, Antioquia, Colombia.

**Keywords:** Preterm birth, Bronchopulmonary dysplasia, Chronic obstructive pulmonary disease, Risk factors, Nascimento prematuro, Displasia broncopulmonar, Doença pulmonar obstrutiva crônica, Fatores de risco

## Abstract

**Objective:**

To determine the incidence, severity, and factors associated with bronchopulmonary dysplasia (BPD) using the Jensen definition in a high-risk preterm infant cohort from a middle-income country.

**Methods:**

A retrospective observational cohort study was conducted, including babies born at <32 weeks of gestational age between 2021 and 2023 at a tertiary neonatal intensive care unit in Colombia. BPD was defined as the need for supplemental oxygen or respiratory support at 36 weeks’ postmenstrual age (PMA) or at discharge. Demographic, perinatal, and clinical variables were obtained from medical records. Factors associated with BPD were evaluated using bivariate analyses and multivariable Poisson regression with robust variance.

**Results:**

Of 395 eligible neonates, 338 were included; 299 (88.4%) survived to 36 weeks PMA or hospital discharge and were evaluable for BPD. The overall incidence of BPD was 76.3%. Most BPD cases were classified as grade 1 (92.1%). Potentially fatal BPD (positive-pressure support or pulmonary vasodilators at 38 weeks PMA) was identified in 4.8% of patients. Overall mortality in the cohort was 13.0%. In the multivariable analysis, BPD was independently associated with extreme prematurity, requirement for invasive mechanical ventilation during hospitalization, diuretic use for fluid overload, documented infection requiring antimicrobial therapy, maternal thyroid disease, and exposure to antenatal corticosteroids, all of which were statistically significant (p<0.05).

**Conclusions:**

BPD incidence was high in this neonatal cohort. Although mild disease predominated, a relevant proportion of severe and potentially fatal cases was observed. The identification of modifiable postnatal factors highlights opportunities to optimize respiratory management, infection prevention, and fluid strategies in similar middle-income settings.

## RESUMO

**Objetivo:**

Determinar a incidência, a gravidade e os fatores associados à displasia broncopulmonar (DBP) utilizando a definição de Jensen em uma coorte de recém-nascidos prematuros de alto risco de um país de renda média.

**Métodos:**

Estudo de coorte observacional retrospectivo, que incluiu recém-nascidos com idade gestacional <32 semanas admitidos entre 2021 e 2023 em uma unidade de terapia intensiva neonatal na Colômbia. A DBP foi definida como a necessidade de oxigênio suplementar às 36 semanas de idade pós-menstrual (IPM) ou na alta hospitalar. Variáveis demográficas, perinatais e clínicas foram obtidas dos prontuários médicos. Os fatores associados à DBP foram avaliados por meio de análises bivariadas e regressão de Poisson multivariável com variância robusta.

**Resultados:**

Dos 395 neonatos elegíveis, 338 foram incluídos; 299 sobreviveram até 36 semanas de IPM ou até a alta hospitalar. A incidência global de DBP foi de 76,3%. A maioria dos casos foi classificada como DBP de grau 1 (92,1%), enquanto a DBP potencialmente fatal (suporte com pressão positiva ou vasodilatadores pulmonares às 38 semanas de IPM) foi identificada em 4,8%. A mortalidade global da coorte foi de 13,0%. Na análise multivariável, a DBP esteve independentemente associada à prematuridade extrema, à necessidade de ventilação mecânica invasiva, ao uso de diuréticos por sobrecarga hídrica, à infecção documentada com necessidade de terapia antimicrobiana, à doença tireoidiana materna e à exposição a corticosteroides antenatais (p<0,05).

**Conclusões:**

A incidência de DBP foi elevada nesta coorte neonatal. Apesar do predomínio de casos leves, observou-se uma proporção relevante de formas graves e potencialmente fatais. A identificação de fatores associados pós-natais modificáveis destaca oportunidades para otimizar o manejo respiratório, a prevenção de infecções e as estratégias de manejo hídrico em contextos semelhantes de países de renda média.

## INTRODUCTION

Bronchopulmonary dysplasia (BPD) is the most common chronic lung complication and a major cause of morbidity in preterm infants.^1,2^ Different definitions classify patients into risk groups to predict respiratory and neurodevelopmental outcomes. The most widely used has been the 2001 National Institutes of Health definition,^3^ based on respiratory support duration and oxygen requirement, although the Jensen definition, based on respiratory support at 36 weeks’ postmenstrual age (PMA), has been increasingly adopted.^4^ This approach has shown greater prognostic accuracy for these outcomes than previous definitions.^1,5,6,7^ Understanding how this classification performs in high-risk units and across diverse socioeconomic settings remains important, particularly where resource limitations may affect disease frequency and severity.

Reported incidence varies widely worldwide, ranging from 10 to 89%, largely depending on gestational age and birth weight, which are the strongest nonmodifiable predictors.^1,8^ Prenatal contributors include intra-amniotic infection, fetal growth restriction, and hypertensive disorders of pregnancy,^1,5,9^ while postnatal factors include prolonged invasive ventilation, patent ductus arteriosus (PDA), infections, transfusions, and positive fluid balance.^7,10,11^

Advances in perinatal care have not reduced BPD incidence; instead, rates have increased due to improved survival of extremely preterm infants.^11^ In Latin America, only one additional Brazilian cohort described the epidemiology of BPD using contemporary definitions, highlighting the need for better characterization of its clinical course in high-risk neonatal units in the region.^12^ This gap is relevant because risk profiles and outcomes may differ from international reports, limiting the applicability of existing predictive models.^2^ Identifying modifiable factors, including ventilatory strategies, infection prevention, and nutritional support, may also improve outcomes. The objective was to determine the incidence, severity, and factors associated with BPD using the Jensen definition in patients born at <32 weeks’ gestational age admitted to a reference neonatal unit in Medellín, Colombia, between 2021 and 2023.

## METHOD

This retrospective cohort study included all preterm neonates born at <32 weeks’ gestation admitted to a tertiary perinatal center in Medellín, Colombia, between 2021 and 2023. Gestational age was selected based on the definition used to classify BPD.^4^ The exclusion criteria were: out-of-hospital birth, transfer before safe discharge, and major congenital anomalies defined by the research team. No prior sample size calculation was performed, as all consecutive eligible patients admitted during the study period were included. The study was conducted and reported in accordance with the Strengthening the Reporting of Observational Studies in Epidemiology (STROBE) Statement.

After protocol approval, data were collected retrospectively from medical records using a standardized Microsoft Excel spreadsheet with predefined variables and validation fields to ensure data quality.

Prenatal and perinatal variables included maternal sociodemographic and clinical characteristics, such as maternal age, Colombian socioeconomic stratification level, urban or rural residence, highest educational level attained, and comorbidities including diabetes, hypertension, preeclampsia, obesity, thyroid disease, and tobacco or psychoactive substance use. Additional variables included prenatal ultrasound findings, cause of prematurity, multiple gestation, gestational age at birth, antenatal corticosteroid exposure, delivery room anthropometric measurements, and neonatal resuscitation data. Standardized international definitions and guidelines were used for fetal growth restriction, anthropometric classification, and neonatal resuscitation, including the INTERGROWTH-21st standards, the International Society of Ultrasound in Obstetrics and Gynecology criteria, and the American Heart Association Neonatal Resuscitation Program guidelines.

Unit procedures and management included respiratory support characteristics, surfactant administration, and pharmacologic therapies such as the DART regimen (Dexamethasone: A Randomized Trial), inhaled corticosteroids, caffeine, pulmonary vasodilators, antibiotics, transfusions, and diuretics administered for persistent positive fluid balance accompanied by clinical evidence of fluid overload. Evaluated comorbidities included anemia requiring transfusion, apnea, severe intraventricular hemorrhage or retinopathy, PDA, and pulmonary hypertension. Extrauterine growth restriction was defined as a decrease of more than one standard deviation from birth weight or a weight below the 10th percentile at discharge or at 36 weeks’ PMA.^13^ Age terminology during the perinatal period followed the American Academy of Pediatrics policy statement.^14^

In-hospital outcomes included BPD incidence, severity classification, and mortality. BPD was diagnosed in infants requiring supplemental oxygen and/or respiratory support at 36 weeks’ PMA or at discharge, and severity was classified according to standardized criteria:^8^

- Grade 1: supplemental oxygen via low-flow nasal cannula at ≤2 L/min^15^
- Grade 2: supplemental oxygen via low-flow nasal cannula at >2 L/min or noninvasive ventilatory support^15^
- Grade 3: invasive mechanical ventilation^15^

Potentially fatal BPD was defined as the presence of positive-pressure respiratory support or the requirement for pulmonary vasodilator therapy at 38 weeks of PMA.^16^

Qualitative variables were expressed as absolute and relative frequencies, while quantitative variables were summarized using the median and interquartile range (IQR), given the non-normal distribution as assessed by the Kolmogorov-Smirnov test.

For the bivariate analysis of factors associated with BPD, patients who died before 36 weeks of PMA were excluded. Variables were selected based on prior evidence in the literature or established pathophysiological plausibility. Quantitative variables were compared using the Mann-Whitney U test, while qualitative variables were analyzed using the chi-square test or Fisher’s exact test, as appropriate. Relative risks (RRs) with corresponding 95% confidence intervals (95%CI) were calculated to compare qualitative variables between patients with and without BPD.

In the multivariable analysis, variables that demonstrated statistically significant associations in the bivariate analysis were included, along with those considered clinically relevant to the development of BPD. A Poisson regression model with robust variance estimation was employed. Adjusted relative risks (aRRs) with their corresponding 95%CI were estimated, and a p-value <0.05 was considered statistically significant. All analyses were performed using IBM Statistical Package for the Social Science (SPSS) software, version 30.

According to Resolution 8430 of 1993 under Colombian Law, this no-risk study did not require informed consent and was approved by the Institutional Health Research Ethics Committee (approval No. 5 of 2024). The investigators ensured adherence to fundamental bioethical principles.

## RESULTS

**Figure 1:**
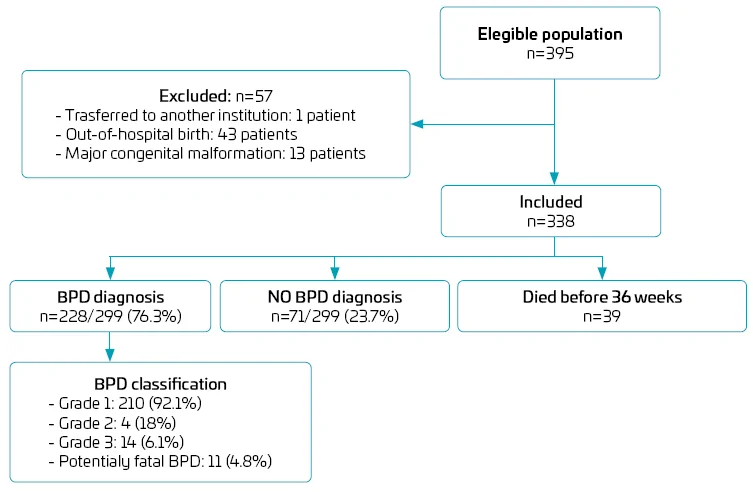
Patient selection process, incidence, and severity classification of bronchopulmonary dysplasia.

Source: Prepared by the authors based on the results obtained in this study.

Among the infants who fulfilled the eligibility criteria (n=395), a total of 338 were included in the study. Of the included infants, 299 (88.4%) were evaluable for BPD at 36 weeks of PMA or at hospital discharge, whereas the remaining 39 died prior to this time point. The incidence of BPD was 76.3% (228/299); the distribution according to severity is shown in Figure 1. A total of 44 deaths (13.0%) were recorded in the entire analyzed population, with a median gestational age of 28+0 weeks (IQR 27+0 to 30+1). Five deaths occurred after 36 weeks of PMA; pulmonary hypertension was the cause in two patients and refractory septic shock in three.

BPD: bronchopulmonary dysplasia.

Regarding maternal characteristics, the median maternal age was 26 years (IQR 21–31). A total of 91 mothers (27.7%) resided in rural dispersed areas, with a median of five prenatal care visits (IQR 3–6). The most frequent maternal comorbidities were severe preeclampsia, chronic or gestational hypertension, and HELLP syndrome (Hemolysis, Elevated Liver enzymes, and Low Platelets) or eclampsia (Table 1). Fetal growth restriction documented on prenatal ultrasound was reported in 75 cases (22.2%), mostly classified as type 1.

**Table 1:** Gestational and perinatal characteristics of neonates born at less than 32 weeks of gestation admitted to the unit between 2021 and 2023.

Variable	BPD n=228, n (%)	No BPD n=71 n (%)	p-value
Severe preeclampsia	57 (25.0)	12 (16.9)	0.157
HELLP syndrome or eclampsia	24 (10.5)	7 (9.9)	0.872
Thyroid disease	18 (7.9)	1 (1.4)	0.053
Tobacco use	2 (0.9)	2 (2.8)	0.240
Prenatal history of IUGR^†^	55 (24.1)	13 (18.3)	0.308
Type 1^‡^	28 (50.9)	10/12 (83.2)	0.040
Type 2^‡^	9 (16.4)	2/12 (16.7)	0.982
Type 3^‡^	17 (30.9)	0	0.020
Type 4^‡^	1 (1.8)	0	0.633
Complete fetal maturation	174 (76.3)	39 (54.9)	0.001
Chorioamnionitis or intra-amniotic infection	18 (7.9)	5 (7.0)	0.814
Gestational age at birth^*^, weeks	29.7 (28.1– 31.0)	31.1 (30.0– 31.9)	<0.001
Extreme prematurity (<28 weeks)	52 (22.8)	2 (2.8)	<0.001
28–32 weeks	176 (77.2)	69 (97.2)	<0.001
Infection or inflammation prematurity	140 (61.4)	49 (69.0)	0.246
Placental dysfunction prematurity	88 (38.6)	22 (31.0)	0.260
Male sex	130 (57.0)	37 (52.1)	0.467
Birth weight, grams^*^	1185 (936–1404)	1495 (1315–1675)	<0.001
<1000	67 (29.4)	3 (4.2)	<0.001
1000–1500	119 (52.2)	33 (46.5)	0.410
>1500	42 (18.4)	35 (49.3)	<0.001
Birth weight percentile^*^	46.3 (20.8–64.9)	50 (34.0–74.1)	0.018
Delivery room intubation	162 (71.1)	34 (47.9)	<0.001
PPV or CPAP in the delivery room	211 (92.5)	60 (84.5)	0.042

^*^Median (interquartile range);

^†^Intrauterine Fetal growth restriction (IUGR) was classified according to Doppler findings as follows: Type I, fetal hemodynamic redistribution without critical flow abnormalities; Type II, absent end-diastolic flow in the umbilical artery and/or reversed flow in the aortic isthmus; Type III, reversed end-diastolic flow in the umbilical artery and/or abnormal ductus venosus pulsatility; and Type IV, advanced fetal compromise with reversed ductus venosus flow and/or pathological cardiotocography;

^‡^In these variables, the information is not complete; therefore, the denominator used to estimate the reported result is specified (n/N).

BPD: bronchopulmonary dysplasia; HELLP: Hemolysis, Elevated Liver enzymes, and Low Platelets; IUGR: intrauterine growth restriction; PPV: positive pressure ventilation; CPAP: continuous positive airway pressure.

Source: Prepared by the authors based on the results obtained in this study.

The median gestational age at birth was 29+4 weeks, and 24.6% of infants were extremely preterm. The etiology of prematurity was classified according to the two major endotypes proposed by Pierro et al.^17^, with most cases attributed to infectious or inflammatory processes, including preterm premature rupture of membranes, cervical insufficiency, and intra-amniotic infection, among others; and the remainder attributed to placental dysfunction, including pregnancy termination due to hypertensive disorders of pregnancy and fetal growth restriction.^17^ Gestational and perinatal characteristics are presented in Table 1.

During their stay in the neonatal intensive unit care, 87.6% received surfactant therapy, most commonly using the INSURE (INtubation, URrfactant administration, and Extubation) technique. Regarding respiratory support, 67.2% required orotracheal intubation at some point, and 16.0% required reintubation during hospitalization. The most frequently used oxygen delivery device was the low-flow nasal cannula in 84.6% of infants. No patient required tracheostomy. Additional information regarding neonatal care is described in Table 2.

**Table 2:** Management and documented comorbidities in the neonatal unit of infants born at <32 weeks of gestation cared for between 2021 and 2023.

Variable	BPD n=228, n (%)	No BPD n=71 n (%)	p-value
Required intubation at any point	167 (73.2)	25 (35.2)	<0.001
Total days of intubation^*^	5 (2–13.5)	2 (1–4)	<0.001
Conventional ventilation	164 (71.9)	21 (29.6)	<0.001
Non-invasive ventilation	162 (71.1)	31 (43.7)	<0.001
Caffeine	188 (82.5)	36 (50.7)	<0.001
Diuretic for evidence of fluid overload	87 (38.2)	2 (2.8)	<0.001
Days of diuretic use*	13 (5–28)	–	<0.001
Systemic corticosteroid for extubation	45 (19.7)	2 (2.8)	<0.001
Inhaled corticosteroid	39 (17.1)	0 (0.0)	<0.001
Pulmonary vasodilator	3 (1.3)	0 (0.0)	0.884
Evidence of any type of infection	70 (30.7)	4 (5.6)	<0.001
Bacteremia	25 (11.0)	0 (0.0)	0.006
Catheter-associated bacteremia	19 (8.3)	0 (0.0)	0.010
Ventilator-associated pneumonia	15 (6.6)	0 (0.0)	0.031
Anemia requiring transfusion	181 (79.4)	23 (32.4)	<0.001
Apneas	162 (71.1)	33 (46.5)	0.005
PDA in the first ten days of life^†^	111/215 (51.6)	19/62 (30.6)	0.004
IVH grade III or higher	7 (3.1)	1 (1.4)	0.792
Pulmonary hypertension near 34 weeks^†^	12/206 (5.8)	2/55 (3.6)	0.403
ROP grade III or higher^†^	6/222 (2.7)	0(0.0)	0.372
Weight: 36 weeks or at discharge, grams*	2065 (1861–2280)	2055(1905–2215)	0.645
Weight percentile*	14.5 (3.3–32.1)	23.1(10.6–36.4)	0.001
Presence of EUGR	203 (89.0)	60 (84.5)	0.309

^*^Median (interquartile range);

^†^In these variables, the information is not complete; therefore, the denominator used to estimate the reported result is specified (n/N).

BPD: bronchopulmonary dysplasia; PDA: patent ductus arteriosus; IVH: intraventricular hemorrhage; ROP: retinopathy of prematurity; EUGR: extrauterine growth restriction

Source: Prepared by the authors based on the results obtained in this study.

Ninety-three infants (27.5%) developed at least one infection during hospitalization, with bloodstream infection being the most common, followed by catheter-associated bloodstream infection. Anemia requiring transfusion, apnea, and evidence of patent ductus arteriosus within the first ten days of life were the main comorbidities associated with prematurity. Extrauterine growth restriction was documented in 78.4% of cases. The remaining variables are presented in Table 2.

In the bivariate analysis, variables that demonstrated statistical significance (p<0.001) included extreme prematurity, low birth weight, intubation in the delivery room, requirement for intubation, total days of intubation, documentation of any type of infection, use of therapeutic antimicrobials, requirement for inhaled corticosteroids, requirement for diuretics due to evidence of fluid overload, and the presence of apnea (Table 3).

**Table 3:** Perinatal factors, management, and comorbidities associated with the diagnosis of bronchopulmonary dysplasia in the treated neonates (n=299).

Factors	n	BPD n (%)	No BPD n (%)	RR (95%CI)	p-value
Antenatal corticosteroid	258	202 (78.3)	56 (21.7)	1.23 (0.9–1.6)	**0.038**
Extreme prematurity	54	52 (96.3)	2 (3.7)	1.34 (1.2–1.5)	**<0.001**
Birth weight, grams	299	1185 (936–1404)^*^	1495 (1327–1673)^*^		**<0.001**
Delivery room intubation	196	162 (82.7)	34 (17.3)	1.29 (1.1–1.5)	**<0.001**
Intubation at any point	192	167 (73.2)	25 (35.2)	1.53 (1.3–1.8)	**<0.001**
Re-intubation at any point	50	47 (94.0)	3 (6.0)	1.29 (1.2–1.4)	**0.001**
Total days of intubation	299	5 (2–13)^*^	2 (1–4)^*^		**<0.001**
Any infection recorded	74	70 (94.6)	4 (5.4)	1.35 (1.2–1.5)	**<0.001**
CAB	19	19 (100.0)	0 (0.0)	1.34 (1.3–1.4)	**0.009**
VAP	15	15 (100.0)	0 (0.0)	1.33 (1.3–1.4)	**0.026**
Bacteremia	25	25 (100.0)	0 (0.0)	1.35 (1.3–1.4)	**0.004**
Therapeutic antimicrobials	71	68 (95.8)	3 (4.2)	1.37 (1.2–1.5)	**<0.001**
Inhaled corticosteroid	39	39 (100.0)	0 (0.0)	1.38 (1.3–1.5)	**<0.001**
Diuretic	89	87 (97.8)	2 (2.2)	1.46 (1.3–1.6)	**<0.001**
Apneas	195	162 (83.1)	33 (16.9)	1.3 (1.1–1.5)	**<0.001**
PDA in the first 10 days of life	130	111 (85.4)	19 (14.6)	1.21 (1.1–1.4)	**0.004**
Weight percentile at 36 weeks or at discharge	299	14.5 (3.4–32.1)^*^	23.1 (10.6–36.4)^*^		**0.001**

^*^Median (interquartile range).

BPD: bronchopulmonary dysplasia; RR: relative risk; CI: confidence interval; CAB: catheter-associated bacteremia; VAP: ventilator-associated pneumonia; PDA: patent ductus arteriosus; Bold: values that are statistically significant.

Source: Prepared by the authors based on the results obtained in this study.

The variables included in the multivariable analysis were extreme prematurity, need for intubation in the delivery room, requirement for reintubation, antimicrobial therapy for documented infection, diuretic use due to fluid overload, antenatal corticosteroid administration, maternal thyroid disease, systemic corticosteroid use for extubation, apnea episodes, and patent ductus arteriosus during the first ten days of life. Variables that remained statistically significant after the analysis are described in Table 4.

**Table 4:** Multivariable analysis of factors associated with bronchopulmonary dysplasia in neonates cared for between 2021 and 2023.

Associated factors	aRR^*^	(95%CI)	p-value
Maternal thyroid disease	1.21	(1.04–1.41)	0.013
Antenatal corticosteroid administration	1.28	(1.03–1.58)	0.026
Extreme prematurity	1.10	(1.00–1.21)	0.050
Required intubation at any time	1.27	(1.04–1.56)	0.026
Requirement for diuretic due to fluid overload	1.25	(1.13–1.39)	<0.001
Required antimicrobials for documented infection	1.16	(1.06–1.28)	0.002

^*^aRR (adjusted risk ratio) estimated using a Poisson regression model with robust variance estimation.

CI: confidence interval.

Source: Prepared by the authors based on the results obtained in this study.

## DISCUSSION

Improved characterization of BPD has led to updated definitions that allow more accurate estimation of its incidence and associated risk factors. In this cohort, the incidence of BPD was 76.3%, which is higher than that reported in most studies. However, the literature demonstrates considerable variability in BPD incidence, likely attributable to differences in the characteristics of the populations studied and the diagnostic criteria applied. In studies including infants born at <32 weeks of gestation and with BPD assessed at 36 weeks of PMA, reported incidences have been 46.4, 50.4, and 29.06%.^11,18,19^ The higher incidence observed in the study cohort may be partially explained by the inclusion of infants requiring supplemental oxygen at discharge prior to 36 weeks of PMA, as proposed by the adopted BPD definition.^4^ This situation is relatively common, as approximately 32.7% of infants born weighing less than 1,500 g are discharged at 35 weeks of PMA.^20^ In this setting, early discharge is facilitated by an established high-risk neonatal follow-up program that enables close outpatient monitoring of clinically stable infants who reach a safe discharge weight and do not meet criteria for continued management in high-dependency neonatal units. Accordingly, the median discharge weight (2060 g; IQR 1870–2240 g) reflects infants with pulmonary vulnerability who remain at high respiratory risk despite meeting discharge criteria.

Regarding severity, the predominance of grade 1 BPD (92.1%) differs from that reported in a Taiwanese cohort, in which grade 2 BPD was the most frequent classification (44%); however, the cohort characteristics were very similar to those of the present study population.^18^ Conversely, grade 3 BPD was more frequent in the study cohort, representing 6.1% of cases, compared with a cohort from the United States in which incidence ranged from 1.3 to 2.2% across different ethnic groups.^21^ This finding is noteworthy considering that the population in that study had lower birth weight and gestational age than the study population.^21^ It is also consistent with the results observed in the subgroup of patients with potentially fatal BPD: in this cohort, 4.8% of patients met this classification, whereas Naples et al. reported an incidence of 13.9 cases per 1000 infants born at <32 weeks of gestational age.^16^ Similarly, reported mortality rates vary across the literature, with the mortality observed in this study being 11.5%, which is lower than that reported in the Argentine cohort (17.1%) and the California cohort (25%).^2,21^ Despite similar population characteristics and classification criteria, the higher mortality in the Argentine cohort may be explained by longer mechanical ventilation and lower birth weight.^2^ In the California cohort, mortality was likely related to the inclusion of smaller and more premature infants.^21^

In this cohort, several maternal antecedents with relevance to the development of BPD were characterized. Up to half of the patients had a maternal history of hypertensive disorders of pregnancy; however, this variable did not remain statistically significant after analysis, possibly due to stratification into severity subgroups (chronic or gestational hypertension, preeclampsia or severe preeclampsia, and HELLP syndrome or eclampsia). In contrast, the literature has documented an association between these disorders and up to a twofold increase in the risk of BPD, a relationship explained by placental dysfunction and its impact on disruption of pulmonary development.^7,9^ The predominant prematurity endotype was inflammation or infection, accounting for 63.9% of cases; however, intra-amniotic infection was confirmed in only 8.3% of patients. The low rate of confirmation, likely related to resource limitations in the study setting, may explain the lack of statistically significant association with BPD, despite existing literature recognizing this antecedent as a relevant risk factor, particularly in the presence of coinfections, with an odds ratio of 5.4 (95%CI 1.7–17) for moderate to severe BPD.^22^ In this cohort, a maternal history of thyroid disease was identified as a statistically significant associated factor. Previous studies have reported a higher prevalence of maternal thyroid disease among infants with BPD; however, it has not been consistently demonstrated as an independent risk factor, and the underlying pathophysiological mechanisms remain unclear. Conversely, the use of antenatal corticosteroids has been shown to reduce neonatal respiratory distress without modifying the incidence of BPD.^6,23^ In the present cohort, antenatal corticosteroid exposure was identified as an associated factor; however, the high rate of administration in both groups suggests that this finding may reflect an obstetric risk marker rather than a direct causal effect, limiting the ability to isolate its true impact on BPD development.

Invasive mechanical ventilation, both in terms of the need for intubation and its duration, was a determining factor for BPD. This finding is consistent with the existing literature, in which invasive ventilation is described as an independent risk factor, and it has been documented that for each additional day of ventilation, the risk of BPD grade 2–3 increases by approximately 8%.^2,8,24^ Likewise, a duration exceeding one week has been independently associated with a significant increase in risk (OR 5.89; 95%CI 2.05–16.8).^10,25^ Furthermore, it is well recognized that delivery room interventions influence the subsequent development of BPD. The requirement for intubation in the delivery room was a significant factor in the bivariate analysis of the present cohort. This observation is supported by findings from a cohort in Saudi Arabia with a population similar to that of the present study, in which this antecedent was significant with an OR of 3.89 (95%CI 1.9–7.6),^4^ as well as by a Japanese cohort of extremely preterm infants that identified invasive ventilation on the first day of life as an associated factor, with an adjusted OR of 2.84 (95%CI 1.5–5.2).^26^

In the study cohort, diuretic use for fluid overload was associated with BPD. This finding is consistent with a Chinese cohort of infants with similar gestational ages, in which older BPD definitions were applied and diuretic use was associated with disease severity.^27^ Likewise, despite using earlier definitions of BPD, Al-Jebawi et al. and Cunha et al. identified the administration of high fluid volumes, particularly during the first week of life, as an independent factor linked to both the development and severity of BPD.^10,28^ In the bivariate analysis, the echocardiographic diagnosis of PDA during the first week of life yielded a statistically significant result; however, imaging studies in later periods were not included for patients in the present cohort, as echocardiography was performed according to the patient’s clinical course, and missing data would have affected the execution of multivariable models. Multiple cohorts have documented that the echocardiographic finding of PDA, independent of its clinical significance, is a factor that increases the risk of BPD in very low birth weight infants.^7,27,28^

In the multivariable analysis, only antimicrobial therapy for documented infections remained significantly associated with BPD. In the bivariate analysis, bacteremia, whether catheter-associated or not, and ventilator-associated pneumonia were also relevant. In other cohorts and meta-analyses, both late- and early-onset sepsis have been identified as independent risk factors for BPD, with ORs of 2.25 (95%CI 1.61–3.13) and 2.52 (95%CI 1.01–6.29), respectively.^7,29^ The need for inhaled corticosteroids likely reflects treatment of established or severe lung disease before 36 weeks of PMA rather than a causal factor in BPD development.^16^ Similarly, apnea likely reflects neurological and respiratory immaturity associated with extreme prematurity rather than an independent risk factor for BPD. Although extrauterine growth restriction was highly prevalent, its frequency was similar between groups and was not significantly associated with BPD. Nevertheless, it remains a modifiable factor linked to BPD occurrence and severity, underscoring the importance of optimizing neonatal nutritional strategies.^30^

This study has several limitations. Its retrospective single-center design may have introduced information bias due to underreporting, heterogeneous documentation, and missing data, although missingness in key variables was low, supporting complete-case analysis. As this approach assumes data were missing completely at random, residual bias cannot be excluded. Additionally, because the study was conducted in a tertiary referral neonatal intensive care unit, findings may not be generalizable to lower-risk populations. Finally, inclusion of infants discharged before 36 weeks of PMA on home oxygen therapy may have affected severity classification; however, this was consistent with the study definition and national consensus statements.^4,15^

Despite these limitations, this study has several strengths. It provides a detailed characterization of a high-risk neonatal population in a middle-income Latin American setting, addressing a persistent gap in regional data. The predominance of grade 1 BPD suggests a shift toward milder disease phenotypes, potentially reflecting advances in neonatal respiratory care despite resource constraints. Although mild disease predominated, a clinically relevant proportion of severe cases persisted. The absence of excess mortality, together with alternative care pathways and structured follow-up, supports the role of optimized perinatal and neonatal management in mitigating disease progression. Importantly, the identification of modifiable postnatal factors highlights opportunities to improve respiratory support, infection prevention, and fluid management. These findings provide locally relevant evidence to inform clinical practice and public health policies aimed at improving outcomes for preterm infants in Colombia and Latin America.

In conclusion, BPD remains a common complication among extremely preterm infants in the study setting. While most cases were mild, the persistence of severe disease and the identification of potentially modifiable risk factors emphasize the need for continued efforts to optimize neonatal care and improve outcomes in this vulnerable population.

## Data Availability

The database that originated the article is available with the corresponding author.
